# Computed tomography findings of a solitary extramedullary plasmacytoma of the spleen: A case report and literature review

**DOI:** 10.3892/ol.2014.2691

**Published:** 2014-11-07

**Authors:** YING WANG, LI YANG, ZI-HUA QIAN, XIU-LIANG ZHU, RI-SHENG YU

**Affiliations:** Department of Radiology, Second Affiliated Hospital, Zhejiang University School of Medicine, Hangzhou, Zhejiang 310009, P.R. China

**Keywords:** splenic neoplasms, solitary extramedullary plasmacytoma, computed tomography

## Abstract

Solitary extramedullary plasmacytoma (EMP) of the spleen is a rare condition. The present study describes the case of a 23-year-old female with an extremely rare solitary EMP of the spleen. Upon examination, the tumor demonstrated unusual and notable multiple-phase spiral computed tomography (CT) findings. The lesion was a solitary, well-defined mass, with areas of variable splenic necrosis and cystic degeneration. Contrast-enhanced CT revealed progressive enhancement of the lesion in the cystic wall, internal septa and solid portion, a finding that has not previously been described. The patient underwent a splenectomy and recovered without complications. No evidence of tumor recurrence has occurred during the past two years of follow-up. To the best of our knowledge, this is the first study to examine the CT findings of a solitary EMP of the spleen. The study aimed to investigate the imaging features of solitary EMP, in particular the multiple-phase spiral CT findings, and raise awareness of the disease to reduce misdiagnoses.

## Introduction

Solitary extramedullary plasmacytoma (EMP) is a rare plasma cell neoplasm that occurs most frequently in head and neck soft-tissue regions, with the nasopharynx, nasal cavity and paranasal sinuses most commonly affected (1). The occurrence of EMP in the spleen is extremely rare. According to a literature search performed in PubMed, a total of six cases of splenic EMP have been previously reported, with only four cases published in English (2–4). However, no studies examining the computed tomography (CT) findings of splenic EMP have been published. The present study investigated an extremely rare case of solitary splenic EMP in a 23-year-old female, examining the imaging features of the lesion, in particular the multiple-phase spiral CT findings, and presented a review of the literature. To the best of our knowledge, this is the first study to examine the CT findings of EMP involving the spleen.

## Case report

A 23-year-old female was admitted to the Second Affiliated Hospital, Zhejiang University School of Medicine (Hangzhou, China) following the discovery of a splenic mass during a routine abdominal ultrasound. The patient had a history of bleeding gums, but not of nausea, dyspnea, fever, abdominal pain or intubation. Furthermore, no positive findings were identified during a physical examination. The results of laboratory tests revealed a hemoglobin level of 129 g/l, a platelet count of 259×10^9^/l and a white blood cell (WBC) count of 8.0×10^9^/l (normal ranges: Hemoglobin level, 113–151 g/l; platelet count, 100–300×10^9^/l; WBC count, 4.0–10.0×10^9^/l). The differential WBC count identified a total of 1.3% eosinophils, 25.3% lymphocytes and 6.2% monocytes monocytes (normal ranges: Eosinophils, 0.0–10.0%; lymphocytes, 20–40%; monocytes, 4.0–12.0%). In addition, the tumor marker levels of carcinoembryonic antigen, α-fetoprotein, carbohydrate antigen (CA)19-9 and CA-125 were normal. An abdominal ultrasound revealed a non-uniform, low echo, 8.4×7.5-cm, round lesion in the spleen. A number of radiological and laboratory tests were performed in order to eliminate systemic plasmacytoma. The full blood count and levels of creatinine, serum calcium and uric acid were within the normal ranges. The chest CT scan was normal, with the absence of any pulmonary lesions or mediastinal lymph nodes. In addition, the emission computed tomography skeletal survey was normal.

**Figure 1:**
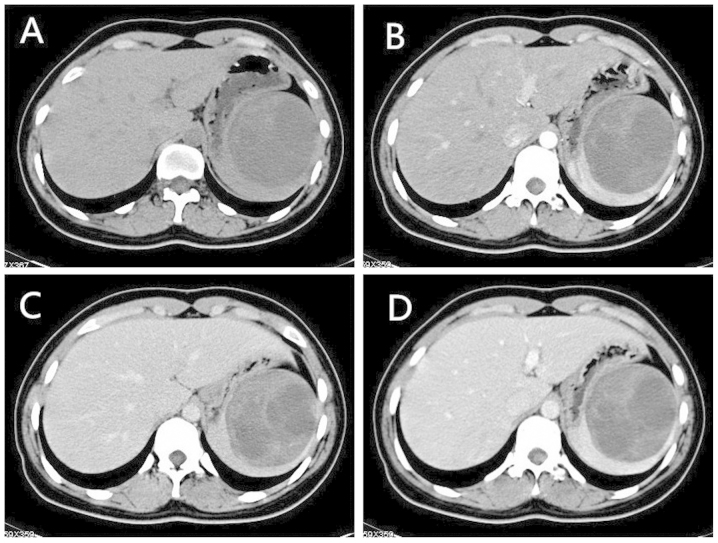
Computed tomography (CT) images. (A) Pre-contrast CT revealing a large, solitary, well-defined mass in the spleen, with variable areas of necrosis and cystic degeneration. Contrast-enhanced CT revealing the progressively-enhanced cystic wall, internal septa and solid portion during the (B) hepatic arterial, (C) portal venous and (D) hepatic parenchymal phases. The areas of necrosis and cystic degeneration were non-enhancing.

The pre-contrast CT revealed that the spleen was enlarged and contained a solitary, well-defined mass, with areas of variable cystic degeneration and necrosis. The mean CT attenuation value of the solid portion was 39 HU, while the values observed for the areas of necrosis and cystic degeneration were 21–23 HU (Fig. 1A). Following a bolus injection of the nonionic contrast agent iopamidol, the cystic wall, internal septa and solid portion of the spleen demonstrated mild enhancement, with a 43 HU attenuation value during the hepatic artery phase. The areas of necrosis and cystic degeneration, however, were non-enhancing (Fig. 1B). During the portal venous and hepatic parenchymal phases, the cystic wall, internal septa and solid portion demonstrated a slight and progressive enhancement, with mean CT attenuation values of 54 HU and 63 HU, respectively (Fig. 1C and D).

**Figure 2:**
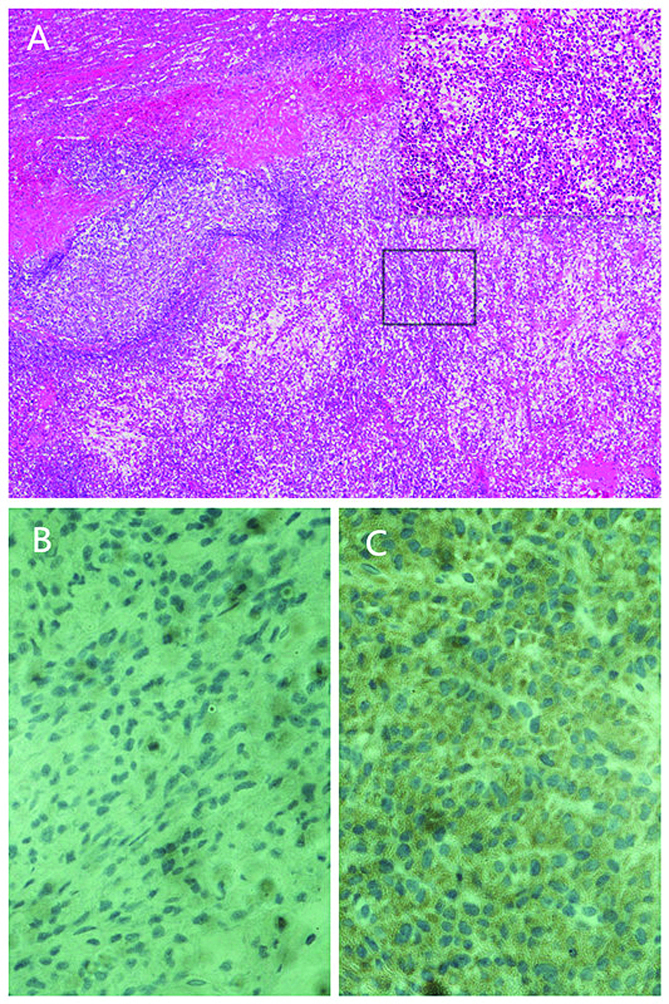
Histological features. (A) Immunohistochemistry revealing the invasion of splenic red pulp tissue by pleomorphic tumor cells (hematoxylin and eosin stain; magnification, ×40). Inset of A: Tumour tissue composed of plasma cells, with eccentrically situated nuclei and a mild degree of nuclear polymorphism (hematoxylin and eosin stain; magnification, ×100). Immunohistochemistry revealing the presence of (B) λ-light chain-positive and (B) κ-light chain-negative cells.

The patient was transferred to the Department of Surgery at the Second Affiliated Hospital, Zhejiang University School of Medicine, where a laparotomy and splenectomy were performed. The resected spleen was enlarged, with lymphoma-like tumors infiltrating the parenchyma. Histopathology revealed a dense monoclonal infiltrate of plasma cells with eccentrically situated nuclei and a mild degree of nuclear polymorphism. In addition to a variety of binuclear cellular forms with scattered mitoses, perinuclear halos were observed in a number of the cells (Fig. 2A). The immunohistochemical analysis revealed that the cells were cluster of differentiation (CD)79A-positive and CD20-negative. Furthermore, the immunohistochemical staining was positive for λ-light chains, but negative for κ-light chains (Fig. 2B and C). These findings were consistent with the diagnostic criteria of plasmacytoma.

Post-operatively, the patient recovered without complications, and no evidence of tumor recurrence has occurred during the past two years of follow-up.

## Discussion

In total, <10% of patients with plasma cell neoplasms present with solitary plasmacytoma (SP). SP is classified according to location as either solitary plasmacytoma of the bone (SPB) or EMP (5). The majority of cases of SPB occur in areas of the axial skeleton, such as the vertebrae and the skull (5), whereas cases of EMP are usually observed in the head and neck. A study consisting of 334 cases of EMP revealed that lesions existing in the upper respiratory tract (in particular the nasal sinuses and pharynx nasalis) accounted for 75% of cases, while the lower respiratory tract represented 4%, the lymph nodes and spleen 6%, the thyroid 3%, the testis 1% and other sites 4% (6).

The median age of patients with either SPB or EMP is 55 years old (5). The overall male to female ratio for SP is 2:1 (7). In the present study, the patient was a 23-year-old female who was considerably below the median age for EMP, and was the youngest reported patient with splenic EMP. Pasch *et al* (8) advocated that trauma may have a potential role in the pathogenesis of SPB affecting young individuals of <30 years old. However, in the present case study, the patient denied the incidence of prior trauma. Furthermore, the symptoms of splenic EMP are often unremarkable. According to the four reported cases of splenic EMP in the English literature (2–4), one patient was admitted to hospital due to right pleuritic chest pain and shortness of breath, and a second experienced chills, night sweats, malaise, a temperature of >38.5°C and a 6-kg loss in weight a month prior to admission. In the present case study, the patient was asymptomatic and discovery of the tumor was incidental. Due to a localized presentation and favorable diagnosis, EMP is distinct from SPB and multiple myeloma (6). Treatment with radiotherapy alone may confer long-term disease-free survival for ~30 and 65% of patients with SPB and EMP, respectively.

Owing to the rarity of the condition, the CT findings of splenic EMP, in particular the radiological manifestations of multiple-phase spiral CT, have not been well-studied. A number of studies investigating the CT findings of EMPs in other organs were identified during the literature search for the present study. One study, which examined a case of pancreatic EMP, demonstrated a large homogeneously-enhancing mass in the region of the pancreatic head (9). A second study revealed a well-demarcated, minimally homogeneously-enhancing, 1.5×1.0×1.2-cm mass in the left arytenoid region of the larynx (10). However, in the present study, CT identified a heterogeneously-enhancing mass with a non-enhancing hypointense area. This observation may be associated with the larger size of the tumor. Upon review of the literature of SPB, the tumor often manifests as a well-defined, ‘punched-out’ lesion with associated soft-tissue masses and bone cortex destruction, with occasional thick-ridging at the periphery, and rare incidences of necrosis or cystic degeneration (11). In the present study, the splenic tumor demonstrated variable areas of necrosis and cystic degeneration, which suggested that EMP may have a different manifestation upon spiral CT than that observed for SPB.

A number of differential diagnoses exist that should be considered when a large, solitary, splenic mass with variable areas of necrosis is detected (12). Malignant tumors, including hematolymphangioma, hemangiosarcoma and lymphoma, and cases of solitary metastasis or abscess, should be ruled out during diagnosis.

The present case study described the case of a 23-year-old female with a large SP of the spleen. To the best of our knowledge, this patient was the youngest of all reported cases. Although EMP of the spleen is extremely rare, and a diagnosis can only be confirmed by pathological examination, the disease should be considered during the differential diagnosis of large, splenic, malignant tumors. In order to reduce the misdiagnosis of EMP, future studies should focus on extending existing clinicopathological knowledge of the disease.
